# Extracellular vesicle-like particles from *Taraxacum mongolicum* suppress non-small cell lung cancer associated with mitochondrial dysfunction

**DOI:** 10.3389/fphar.2026.1802298

**Published:** 2026-04-17

**Authors:** Wei Peng, Renyi Yang, Jincheng Tang, Xiaolan Jian, Huazhong Wang

**Affiliations:** 1 Hunan Provincial Hospital of Integrated Traditional Chinese and Western Medicine, Hunan Academy of Chinese Medicine, Changsha, Hunan, China; 2 Hunan University of Chinese Medicine, Changsha, Hunan, China

**Keywords:** extracellular vesicle-like particles, mitochondrial dysfunction, non-small cell lung cancer, oxidative phosphorylation, Taraxacum mongolicum

## Abstract

**Introduction:**

Plant-derived extracellular vesicle-like particles (EVLPs) are emerging as natural, orally deliverable nanomaterials, but their antitumor activity and mitochondrial mechanisms in non-small cell lung cancer (NSCLC) remain insufficiently defined.

**Methods:**

Taraxacum mongolicum-derived EVLPs (TM-EVLPs) were isolated by enzymatic digestion and differential ultracentrifugation and characterized by nanoparticle tracking analysis, transmission electron microscopy, and protein profiling. Cellular uptake and antitumor effects were evaluated in A549 and H1975 cells using proliferation, migration, invasion, epithelial–mesenchymal transition, and mitochondrial function assays. In vivo efficacy and safety were assessed in an A549 xenograft model after oral TM-EVLP administration.

**Results:**

DiI-labeled TM-EVLPs showed clear cell-associated signals after incubation and washing. TM-EVLPs inhibited NSCLC cell viability, DNA synthesis, clonogenicity, migration, and invasion, with more consistent effects at higher concentrations, and increased E-cadherin but decreased N-cadherin expression. TM-EVLP treatment was associated with mitochondrial membrane potential depolarization, increased mitochondrial reactive oxygen species, reduced ATP production, disrupted cristae ultrastructure, and decreased oxidative phosphorylation complex subunits. In xenograft-bearing mice, oral TM-EVLPs (5–20 mg/kg, 15 days) reduced tumor growth, decreased Ki-67 and PCNA staining, increased TUNEL positivity, recapitulated mitochondrial impairment in tumors, and caused no overt histological injury in major organs.

**Discussion:**

TM-EVLPs suppress NSCLC malignant phenotypes in association with constrained mitochondrial bioenergetics and increased oxidative stress, supporting a mitochondria-centered stress-linked working model.

## Introduction

Lung cancer remains a major global cause of cancer mortality, and non-small cell lung cancer (NSCLC) accounts for approximately 85% of cases (Duma et al., 2019; Bray et al., 2024). Although platinum-based chemotherapy, targeted therapies, and immunotherapy have improved outcomes for selected patients, durable benefit is limited by interpatient heterogeneity and frequent primary or acquired resistance (Wang H. et al., 2025). These challenges underscore the need for interventions that exploit actionable vulnerabilities in NSCLC.

Mitochondria sit at the intersection of energy production and redox homeostasis and are increasingly recognized as a tractable liability under therapeutic and microenvironmental stress (Parma et al., 2022; Salaroglio et al., 2022). NSCLC cells can rely on oxidative phosphorylation (OXPHOS) to sustain proliferation and adapt to stress, and perturbing mitochondrial bioenergetics can amplify oxidative stress while limiting the metabolic support required for malignant phenotypes (Chen et al., 2024). Against this background, mitochondria-centered strategies have gained attention as a complementary approach to restrain tumor growth and aggressiveness.

In recent years, plant-derived extracellular vesicle-like particles (EVLPs) have been increasingly recognized as nanoscale carriers of natural-product cargo, including lipids, proteins, nucleic acids, and plant metabolites, with defined tissue distribution and cellular uptake properties, thereby providing new opportunities for disease intervention (Chu et al., 2024; Zhao B. et al., 2024). *Taraxacum mongolicum* is a widely used traditional botanical drug, and pharmacological studies have reported anti-inflammatory, antioxidant, and antitumor activities that are associated with multiple metabolite classes (e.g., triterpenoids, phenolic acids, and flavonoids). For example, the triterpenoid metabolite taraxasterol has been reported to suppress NSCLC growth and modulate apoptosis-related processes and the tumor microenvironment (Lu et al., 2022; Zhang et al., 2022). Notably, EVLPs derived from *Taraxacum mongolicum* (TM-EVLPs) have shown bioactivity in inflammation-related models (Sun et al., 2025). In addition, prior LC–MS/MS profiling has established a reproducible chemical fingerprint for TM-EVLP preparations and suggested enrichment of phenylpropanoid/phenolic-acid–related metabolites together with flavonoid-class compounds, providing compositional context for preparation quality control and downstream bioactivity assessment. This prior chemical fingerprint provides complementary compositional context for TM-EVLP preparation identity and supports the interpretation of batch-to-batch consistency in the present work. However, their antitumor efficacy in NSCLC and the extent to which they perturb mitochondrial structure and bioenergetics remain insufficiently defined.

On the basis of this background, TM-EVLPs were investigated with a focus on their antitumor potential in NSCLC and the underlying mitochondria-related mechanisms. First, procedures for TM-EVLP isolation and characterization were established to define basic physicochemical properties and ensure batch consistency. Next, the association of TM-EVLPs with NSCLC cells was evaluated *in vitro*, and their effects on malignant phenotypes, including proliferation, migration, and invasion, were assessed. Epithelial–mesenchymal transition (EMT)-related molecular markers were examined in parallel to capture accompanying features of phenotypic changes. At the mechanistic level, evidence was generated from mitochondrial function and mitochondrial morphology to evaluate the impact of TM-EVLPs on mitochondrial homeostasis. Furthermore, antitumor efficacy was validated in a xenograft model following oral administration, and safety was assessed by histological evaluation of major organs. Collectively, these experiments provide experimental evidence supporting TM-EVLPs as a potential intervention strategy for NSCLC.

## Materials and methods

### Materials

Dried botanical drug of *Taraxacum mongolicum* Hand.-Mazz. was purchased from Xinlvyuanshan Specialty Products Co., Ltd. (Heilongjiang Province, China) and authenticated by the Herbal Medicine Unit of the Affiliated Hospital of the Hunan Academy of Chinese Medicine, meeting the standards of the Chinese Pharmacopoeia (2025 edition). To improve material traceability, procurement and authentication records of the source material were archived by the corresponding laboratory for editorial review upon request. All key reagents, kits, consumables, and instruments (including manufacturers and catalog numbers) are provided in Supplementary Table S1, and all antibodies are listed in Supplementary Table S2. Unless otherwise stated, experiments were performed according to the manufacturers’ instructions.

### Isolation of TM-EVLPs

After removal of surface impurities, dried *Taraxacum mongolicum* Hand.-Mazz. was cut into small pieces, rinsed with distilled water, and blotted dry. TM-EVLPs were isolated by mild enzymatic digestion as previously described (Zhao et al., 2023). Briefly, plant tissues were incubated in a digestion buffer containing 4% cellulase, 2% pectinase, and 0.6 mol/L mannitol (pH 5.8) at 50 °C for 6 h, followed by differential centrifugation to remove debris (16,000 × g for 1 h; 2,000 × g for 30 min at 4 °C; and 10,000 × g for 45 min at 4 °C). The clarified supernatant was passed through a 0.45-µm membrane and ultracentrifuged at 100,000 × g for 70 min at 4 °C. The pellet was washed once by resuspension in pre-chilled phosphate-buffered saline (PBS) and re-ultracentrifuged at 100,000 × g for 70 min at 4 °C. The final TM-EVLP pellet was resuspended in pre-chilled PBS, aliquoted, and stored at 4 °C for short-term use or −80 °C for long-term storage. All procedures were performed under aseptic conditions using sterile buffers and consumables, and the final TM-EVLP suspension was sterile-filtered through a 0.22-µm membrane before aliquoting for downstream *in vitro* and *in vivo* use. For process transparency, each preparation batch was generated from 500 g of dried botanical material digested in 5.0 L of enzyme-containing buffer, and the final washed TM-EVLP pellet was resuspended in 8.0 mL sterile PBS. Under this preparation scale, the herb-to-final suspension ratio was 62.5 g raw drug per mL final suspension.

### Characterization and quantification of TM-EVLPs

Nanoparticle tracking analysis (NTA) was used to determine the size distribution and particle concentration of TM-EVLPs. Samples were diluted in pre-chilled PBS to an appropriate concentration and analyzed using a ZetaView system (Particle Metrix, Germany); measurements were performed in triplicate. Transmission electron microscopy (TEM) was performed to visualize vesicle morphology. TM-EVLPs were adsorbed onto carbon-coated copper grids (1–2 min), negatively stained with 2% uranyl acetate, air-dried, and imaged at an accelerating voltage of 80–120 kV. For protein profiling, TM-EVLPs were mixed with sodium dodecyl sulfate polyacrylamide gel electrophoresis (SDS-PAGE) sample loading buffer, denatured, separated by SDS-PAGE, and visualized by Coomassie brilliant blue R-250 staining (Beyotime, P0003S). TM-EVLP protein concentration was quantified using the bicinchoninic acid (BCA) method with a BCA Protein Assay Kit (Beyotime, P0010) according to the manufacturer’s instructions; concentrations were calculated from a standard curve and used to evaluate batch-to-batch consistency. For source-relevant compositional context, we referred to our previously established LC–MS/MS fingerprinting dataset of TM-EVLPs, which showed a stable metabolite pattern enriched mainly in phenylpropanoid/phenolic-acid–related constituents together with flavonoid-class compounds.

### Cell culture and treatment

A549 cells were cultured in Ham’s F-12K medium supplemented with 10% fetal bovine serum (FBS) and 1% penicillin–streptomycin solution (P/S), whereas H1975 cells were maintained in RPMI-1640 medium supplemented with 10% FBS and 1% P/S. Cells were incubated at 37 °C in a humidified atmosphere with 5% CO_2_ and routinely passaged under standard conditions. Only cells in a stable growth phase after recovery were used for experiments, and passage numbers were kept below 20.

TM-EVLP working solutions were prepared according to particle number concentration. Frozen TM-EVLPs were thawed slowly at 4 °C, gently mixed, and diluted with pre-chilled PBS to the desired concentrations while maintaining an identical dosing volume across groups. Range-finding CCK-8 assays were first performed across 5.75 × 10^10^–9.2 × 10^11^ particles/mL; 2.3 × 10^11^ particles/mL was the lowest concentration that produced a reproducible reduction in viability in both A549 and H1975 cells and was therefore selected as the baseline working concentration. Accordingly, 2.3 × 10^11^, 4.6 × 10^11^, and 9.2 × 10^11^ particles/mL (1×, 2×, and 4× of the baseline) were used for subsequent *in vitro* experiments. A synthetic “blank” lipid nanoparticle control was not included because it would not be compositionally or biophysically matched to TM-EVLPs and could introduce confounding lipid effects; therefore, PBS was used as the vehicle control, and TM-EVLPs were washed by ultracentrifugation to minimize carryover of non-vesicular lipids. Cisplatin (purity ≥98%) was included as a clinically established cytotoxic benchmark at a final concentration of 5 μM for *in vitro* assays (treatment duration followed the corresponding assay protocol as described below), to contextualize the magnitude of TM-EVLP effects rather than to imply mechanistic equivalence. Working solutions were prepared in sterile PBS in a biosafety cabinet and used immediately after preparation to minimize microbial contamination.

### Cellular uptake assay

To evaluate cellular uptake of TM-EVLPs by NSCLC cells, TM-EVLPs were labeled with 1,1′-dioctadecyl-3,3,3′,3′-tetramethylindocarbocyanine perchlorate (DiI) working solution (Beyotime, C1036) for 30 min in the dark. Unbound dye was removed by ultracentrifugation, and the pellet was resuspended in sterile PBS; this washing step was repeated once to minimize residual free dye and background fluorescence. A549 and H1975 cells were seeded on sterile glass coverslips placed in 24-well plates and treated at 60%–70% confluence with DiI-labeled TM-EVLPs (9.2 × 10^11^ particles/mL); control cells received an equal volume of PBS. After incubation, cells were washed three times with pre-chilled PBS to remove unbound material, fixed with 4% paraformaldehyde for 15 min at room temperature, and counterstained with 4′,6-diamidino-2-phenylindole (DAPI) for 10 min. Samples were mounted with an anti-fade mounting medium and imaged using a confocal laser scanning microscope. DiI fluorescence was excited at 561 nm and DAPI was excited at 405 nm. Images were collected with identical acquisition settings across groups within each experiment. Fluorescence images were analyzed in ImageJ by measuring mean red fluorescence intensity per cell after background subtraction from at least three randomly selected fields per condition. DiI fluorescence (red) indicated TM-EVLP–associated signals after incubation and washing, and DAPI fluorescence (blue) marked nuclei.

### Proliferation-related assays

A Cell Counting Kit-8 (CCK-8) assay was performed to evaluate cell viability. A549 and H1975 cells were seeded into 96-well plates at 5 × 10^3^ cells/well, allowed to attach, and treated with TM-EVLPs at the indicated particle concentrations (5.75 × 10^10^, 1.15 × 10^11^, 2.3 × 10^11^, 4.6 × 10^11^, and 9.2 × 10^11^ particles/mL); control cells received an equal volume of PBS. After 24 h, CCK-8 reagent (Beyotime, C0038) was added and incubated for 2 h in the dark, and absorbance was measured at 450 nm to calculate relative viability.

A 5-ethynyl-2′-deoxyuridine (EdU) incorporation assay was used to assess DNA synthesis. A549 and H1975 cells were seeded into 24-well plates at 5 × 10^4^ cells/well and treated for 24 h as indicated. EdU labeling was performed using an EdU working solution (Beyotime, C0071L) according to the manufacturer’s instructions, followed by fixation with 4% paraformaldehyde for 15 min and permeabilization with 0.3% Triton X-100 for 10 min at room temperature. The Click reaction was carried out in the dark for 30 min, nuclei were counterstained with DAPI for 5 min, and images were acquired using a fluorescence microscope. The percentage of EdU-positive cells was quantified to estimate proliferative activity. As cisplatin primarily exerts cytotoxicity through DNA damage, comparisons to cisplatin are provided to contextualize effect size rather than to imply a shared mechanism.

A colony formation assay was performed to evaluate long-term proliferative capacity. A549 and H1975 cells were seeded into 6-well plates at 800 cells/well and treated for 24 h as indicated, after which cells were maintained in fresh complete medium with medium changes every 3 days until visible colonies formed. Colonies were fixed with 4% paraformaldehyde for 15 min and stained with 0.1% crystal violet for 20 min. Colonies containing ≥50 cells were counted, and plating efficiency was calculated.

### Migration and invasion assays

A wound healing assay was performed to evaluate migratory capacity. A549 and H1975 cells were seeded into 6-well plates at 5 × 10^5^ cells/well and grown to approximately 90% confluence. A linear wound was generated using a sterile 200 µL pipette tip, and detached cells were removed by washing with pre-chilled PBS. Cells were then incubated with the indicated treatment media, and images of the same fields were captured at 0 h, 24 h, and 48 h using an inverted microscope. Wound areas were quantified using ImageJ software, and migration rates were calculated accordingly.

A Transwell invasion assay was used to assess extracellular matrix invasion. Transwell inserts (24-well format) were pre-coated with diluted Matrigel, and treated cells were seeded into the upper chambers. Complete medium containing 10% FBS was added to the lower chambers as a chemoattractant. After 24 h of incubation at 37 °C, cells on the lower surface were fixed with 4% paraformaldehyde for 15 min and stained with 0.1% crystal violet for 20 min. Five random fields per insert were imaged, and invaded cells were counted to evaluate invasive capacity.

### Mitochondrial function assays

Mitochondrial membrane potential (ΔΨm) was assessed using a JC-1 fluorescent probe. A549 and H1975 cells were seeded into 6-well plates at 2 × 10^5^ cells/well, allowed to attach overnight, and treated as indicated. Cells were then incubated with JC-1 working solution (Beyotime, C2006) at 37 °C for 20 min in the dark. After washing with the kit-provided buffer, cells were maintained in phenol red-free medium, imaged, and the red/green fluorescence ratio was quantified using ImageJ to reflect ΔΨm.

Mitochondrial reactive oxygen species were measured using MitoSOX Red. Following seeding and treatment as above, cells were incubated with MitoTracker Green working solution (Beyotime, C1048) for 30 min and then with MitoSOX Red working solution (Beyotime, S0061M) for 10 min, both in the dark. Nuclei were counterstained with Hoechst 33342 (Beyotime, C1025) for 10 min, and cells were mounted using an anti-fade mounting medium. Multi-channel images (Hoechst, MitoTracker Green, and MitoSOX Red) were acquired using a confocal laser scanning microscope under identical exposure settings across groups. MitoSOX Red fluorescence intensity was quantified within mitochondrial regions with reference to the MitoTracker Green signal to evaluate changes in mitochondrial reactive oxygen species.

Cellular adenosine triphosphate (ATP) levels were determined using a luminescence-based ATP assay kit (Beyotime, S0026). A549 and H1975 cells were seeded into 96-well plates at 8 × 10^3^ cells/well, allowed to attach, and treated as indicated. At the indicated time points, cells were lysed and mixed with the ATP detection working solution according to the manufacturer’s instructions, and luminescence was recorded using a microplate reader. ATP levels were calculated from a standard curve and used to compare cellular energy status among groups.

### TEM for mitochondrial ultrastructure

TEM was performed to assess mitochondrial ultrastructural alterations in NSCLC cells after TM-EVLPs treatment. Following treatment, A549 and H1975 cells were harvested and pelleted, fixed in 2.5% glutaraldehyde at 4 °C for 2 h, and post-fixed in 1% osmium tetroxide at room temperature for 1 h. Samples were dehydrated through a graded ethanol series, transitioned into acetone, and embedded in epoxy resin, followed by polymerization at 60 °C for 48 h. Ultrathin sections (60–80 nm) were prepared and contrast-stained with 2% uranyl acetate and lead citrate. Images were acquired at an accelerating voltage of 80–120 kV.

### Western blot analysis

Western blotting was performed to determine the expression levels of target proteins in cells and tumor tissues. After treatment, cells were washed with pre-chilled PBS and lysed in radioimmunoprecipitation assay (RIPA) buffer supplemented with protease inhibitors on ice for 30 min. Lysates were cleared by centrifugation at 12,000 × g for 15 min at 4 °C, and the supernatants were collected as total cellular protein. To assess OXPHOS-related proteins, mitochondrial fractions were additionally prepared from cells using a mitochondria isolation kit (Beyotime, C3601) according to the manufacturer’s instructions. Tumor tissues were minced on ice, and mitochondrial proteins were extracted using a tissue mitochondria isolation kit (Beyotime, C3606) following the manufacturer’s instructions. Protein concentration was quantified using the BCA method, and equal amounts of total protein or mitochondrial protein were loaded.

Protein samples were mixed with SDS-PAGE sample loading buffer, denatured at 95 °C for 5 min, separated by SDS-PAGE, and transferred onto polyvinylidene difluoride (PVDF) membranes. Membranes were blocked with 5% non-fat milk for 1 h at room temperature and incubated with primary antibodies at 4 °C overnight, followed by horseradish peroxidase (HRP)-conjugated secondary antibodies for 1 h at room temperature. Antibodies against EMT-related proteins (E-cadherin and N-cadherin) and the corresponding loading control (β-actin), as well as a Total OXPHOS antibody cocktail recognizing ATP5A, UQCRC2, MTCO1, SDHB, and NDUFB8 with VDAC1 as the mitochondrial loading control, are listed in Supplementary Table S2. Membranes were washed with Tris-buffered saline with Tween 20 (TBST) and developed using enhanced chemiluminescence.

Band intensities were quantified using ImageJ software. E-cadherin and N-cadherin were normalized to β-actin, and ATP5A, UQCRC2, MTCO1, SDHB, and NDUFB8 were normalized to VDAC1. Relative expression levels were calculated using the control group as the reference.

### Animal experiments

All animal procedures were approved by the Ethics Committee of the Medical Experimental Animal Center, Hunan Academy of Chinese Medicine (approval No. SB2025-0190). Female BALB/c nude mice (6 weeks old, 18–22 g) were purchased from Hunan Silaikejingda Experimental Animal Co., Ltd. (Changsha, China) and housed under specific pathogen-free (SPF) conditions with controlled temperature and humidity, a 12-h light/dark cycle, and *ad libitum* access to food and water.

For xenograft establishment, A549 cells were harvested, resuspended in serum-free medium, and mixed with Matrigel at a 1:1 (v/v) ratio. Each mouse was subcutaneously inoculated in the right axilla with 5 × 10^6^ cells in 100 µL. When tumor volumes reached approximately 80–100 mm^3^, mice were randomized into five groups with comparable baseline tumor volumes (n = 6 per group): control, TM-EVLPs low dose, TM-EVLPs medium dose, TM-EVLPs high dose, and cisplatin. TM-EVLPs were administered by oral gavage once daily for 15 consecutive days at 5, 10, and 20 mg/kg (based on total vesicle protein content) for the low-, medium-, and high-dose groups, respectively. Dose levels were defined *a priori* to span a low-to-high window for dose–response testing of an enriched vesicle preparation and were selected within the protein-based extracellular vesicle dosing range commonly used in preclinical studies, while modeling pharmacological exposure rather than routine dietary intake of Taraxacum preparations (Gupta et al., 2021). TM-EVLPs were diluted in PBS and dosed at 10 mL/kg according to body weight; control mice received an equal volume of PBS. For oral gavage, TM-EVLP suspensions were freshly prepared in sterile PBS according to body weight and administered on the day of preparation. Cisplatin was administered via intraperitoneal injection (5 mg/kg, once every 3 days) as a clinical efficacy benchmark for antitumor comparison, rather than as a mechanistic comparator.

Tumor length (L) and width (W) were measured every 3 days, and tumor volume was calculated as V = (L × W^2^)/2. Body weight and general condition were recorded throughout the study. After the final administration, mice were euthanized; tumors were excised, weighed, and photographed. Major organs were collected. Tumor and organ tissues were either fixed in 4% paraformaldehyde for paraffin embedding/sectioning or snap-frozen in liquid nitrogen and stored at −80 °C for subsequent analyses, including protein assays.

### Histology, immunohistochemistry, and TUNEL staining

Tumor tissues and major organs (liver, heart, spleen, lung, and kidney) were collected, fixed in 4% paraformaldehyde for 24 h, processed routinely, paraffin-embedded, and sectioned at approximately 4 µm. Sections were deparaffinized in xylene and rehydrated through graded ethanol before staining. Hematoxylin and eosin (H&E) staining was performed to evaluate tumor histomorphology and to assess histological safety in major organs. Briefly, sections were stained with hematoxylin (Beyotime, C0105S), blued under running water, counterstained with eosin, dehydrated, cleared, and mounted.

Immunohistochemistry (IHC) was performed to assess Ki-67, proliferating cell nuclear antigen (PCNA), E-cadherin, and N-cadherin expression in tumor tissues. After deparaffinization and rehydration, heat-induced antigen retrieval was conducted using citrate buffer. Endogenous peroxidase activity was quenched with 3% hydrogen peroxide for 10 min, followed by blocking for 30 min at room temperature. Sections were incubated with primary antibodies at 4 °C overnight and then with a HRP-conjugated secondary antibody, followed by 3,3′-diaminobenzidine (DAB) development and hematoxylin counterstaining. Images were acquired using a light microscope. IHC signals were quantified using ImageJ as the percentage of positively stained area under identical threshold settings.

Apoptosis in tumor tissues was evaluated using terminal deoxynucleotidyl transferase dUTP nick end labeling (TUNEL). Sections were processed according to the instructions of a TUNEL assay kit (Beyotime, C1170M), counterstained with DAPI, and mounted. TUNEL-positive signals and DAPI-stained nuclei were imaged using a fluorescence microscope, and the percentage of TUNEL-positive cells was calculated.

### Immunofluorescence staining and ROS detection in tumor tissues

Immunofluorescence staining was performed to assess mitochondria-associated proteins in tumor tissues. Paraffin-embedded tumor sections were deparaffinized, rehydrated, and subjected to heat-mediated antigen retrieval in citrate buffer. After cooling, sections were permeabilized with 0.3% Triton X-100 for 10 min and blocked with 5% bovine serum albumin (BSA) for 60 min at room temperature. Sections were incubated with primary antibodies against MTCO1 and TOMM20 at 4 °C overnight, followed by fluorophore-conjugated secondary antibodies for 60 min at room temperature in the dark. Nuclei were counterstained with DAPI for 10 min. Sections were mounted using an anti-fade mounting medium and imaged with a confocal laser scanning microscope. Fluorescence signal intensities were quantified using ImageJ.

Tumor ROS levels were evaluated using dihydroethidium (DHE) fluorescence staining. Fresh tumor tissues were embedded in optimal cutting temperature (OCT) compound and cryosectioned at approximately 8 µm. Sections were incubated with DHE working solution (Beyotime, S0064S) at 37 °C for 30 min in the dark, washed with PBS, counterstained with DAPI for 5 min, and mounted. Fluorescence images were captured immediately using a fluorescence microscope under identical acquisition settings across groups, and mean fluorescence intensity was quantified using ImageJ.

### Statistical analysis

Statistical analyses were performed using R software (version 4.5.1). Quantitative data are presented as mean ± SEM. The reported n indicates independent experiments (biological replicates). For two-group comparisons, the Mann–Whitney U test (Wilcoxon rank-sum test) was used. For comparisons involving more than two groups, Kruskal–Wallis tests were applied separately within each cell line, followed by Dunn’s *post hoc* tests with Holm adjustment; *post hoc* comparisons were prespecified and restricted to comparisons against the control group. For wound-healing assays, group comparisons were conducted at prespecified time points using the same nonparametric approach. All tests were two-sided, and P < 0.05 was considered statistically significant.

## Results

### Isolation and characterization of TM-EVLPs

**FIGURE 1:**
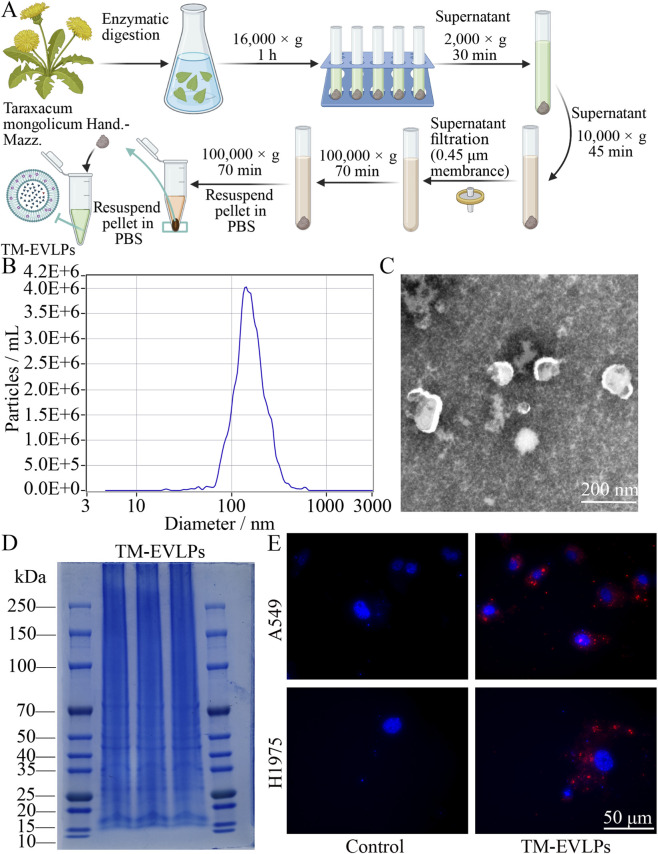
Isolation, characterization, and cellular uptake of TM-EVLPs. **(A)** Schematic of TM-EVLPs isolation. **(B)** Nanoparticle tracking analysis profile of TM-EVLPs. **(C)** Transmission electron microscopy image of TM-EVLPs morphology. **(D)** Coomassie brilliant blue–stained SDS–PAGE showing the protein profile of TM-EVLPs. **(E)** Representative fluorescence microscopy images showing cell-associated signals of DiI-labeled TM-EVLPs (red) in A549 and H1975 cells, with nuclei counterstained by DAPI (blue). TM-EVLPs, *Taraxacum mongolicum*-derived extracellular vesicle-like particles.

TM-EVLPs were obtained using an enzymatic digestion and differential ultracentrifugation workflow (Figure 1A), followed by basic characterization of size distribution, morphology, and protein cargo. NTA showed that TM-EVLPs displayed a single dominant peak distribution, with particle sizes mainly within the nanoscale range. The median diameter based on number distribution was 150.1 nm, the mean diameter was 164.7 nm, and the modal diameter was approximately 143.0 nm (Figure 1B). Morphologically, TEM revealed vesicle-like nanostructures with clear boundaries, and the observed size was consistent with the NTA results (Figure 1C). Coomassie brilliant blue staining further demonstrated a detectable overall protein banding pattern in TM-EVLPs samples (Figure 1D), indicating the presence of protein cargo. BCA quantification showed a protein concentration of 1.9 mg/mL, supporting subsequent sample preparation and batch consistency control. Collectively, these multidimensional characterization results confirmed that TM-EVLP preparations suitable for downstream functional studies were successfully obtained.

### Cellular uptake of TM-EVLPs and inhibition of proliferation *in vitro*

To validate the interaction between TM-EVLPs and NSCLC cells, fluorescence labeling experiments showed discrete DiI signals distributed in the cellular region of A549 and H1975 cells after incubation with DiI-labeled TM-EVLPs, whereas only minimal background fluorescence was observed in the PBS-treated control group (Figure 1E). These observations supported that TM-EVLPs were associated with NSCLC cells following incubation and washing.

**FIGURE 2:**
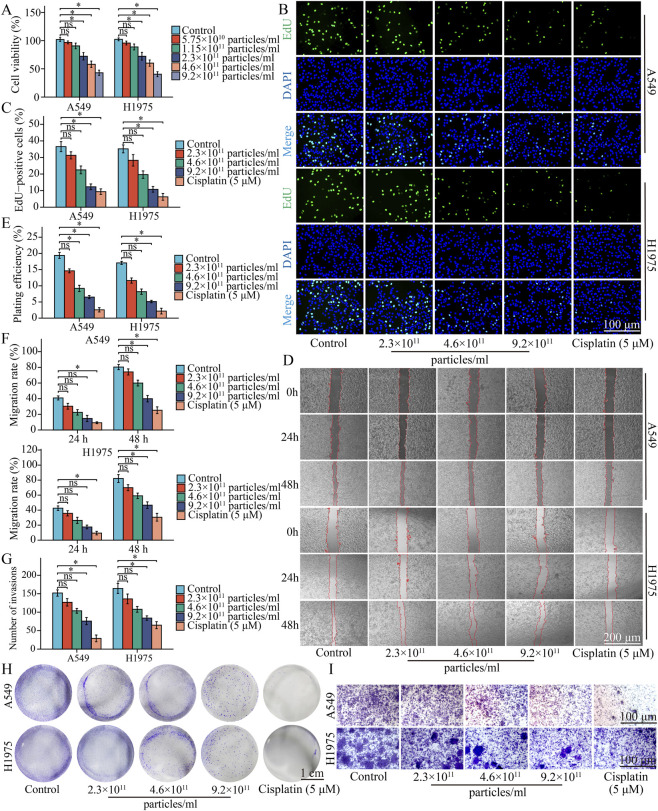
TM-EVLPs suppress NSCLC cell proliferation, clonogenicity, migration, and invasion *in vitro*. **(A)** Cell viability of A549 and H1975 cells after TM-EVLPs treatment at the indicated particle concentrations. **(B)** Representative EdU incorporation images in A549 and H1975 cells treated with TM-EVLPs or cisplatin (5 μM), with DAPI counterstaining. **(C)** Quantification of EdU-positive cells. **(D)** Representative wound-healing images at the indicated time points following TM-EVLPs treatment or cisplatin (5 μM). **(E)** Quantification of plating efficiency from colony formation assays. **(F)** Quantification of wound-healing migration rate. **(G)** Quantification of invaded cells in Transwell invasion assays. **(H)** Representative colony formation images. **(I)** Representative Transwell invasion images. TM-EVLPs, *Taraxacum mongolicum*-derived extracellular vesicle-like particles; NSCLC, non-small cell lung cancer; EdU, 5-ethynyl-2′-deoxyuridine; DAPI, 4′,6-diamidino-2-phenylindole. *P < 0.05, ns, not significant. Data are presented as mean ± SEM (n = 3 independent experiments).

To balance detectable biological effects with experimental feasibility, a CCK-8 assay was first used to screen an appropriate concentration range. Compared with the control group, no significant changes in cell viability were observed at lower concentrations (5.75 × 10^10^ particles/mL and 1.15 × 10^11^ particles/mL) in either A549 or H1975 cells. A decreasing trend in viability emerged when the concentration increased to 2.3 × 10^11^ particles/mL or higher (Figure 2A). The two higher concentrations were defined as 2-fold and 4-fold multiples of this baseline to enable a structured dose–response evaluation. On this basis, 2.3 × 10^11^ particles/mL was selected as the baseline working concentration for subsequent *in vitro* experiments, and a dose gradient of 2.3 × 10^11^, 4.6 × 10^11^, and 9.2 × 10^11^ particles/mL was applied to systematically evaluate the impact of TM-EVLPs on proliferative phenotypes.

In proliferation assays, EdU incorporation showed an overall dose-associated reduction in the proportion of EdU-positive cells in A549 and H1975 cells after TM-EVLPs treatment (Figures 2B,C), with more consistent inhibitory effects observed at the high concentration, and a decreasing trend at the medium concentration. At the highest concentration, the decrease in EdU positivity approached the magnitude observed in the cisplatin group (5 μM). Colony formation assays further supported these findings, showing diminished clonogenic capacity in both cell lines after TM-EVLPs treatment, as reflected by reduced colony density. Quantitative analysis showed an overall decreasing trend in clonogenic capacity across the dose range, with the most consistent inhibition at the high concentration (Figures 2E,H). Collectively, these results demonstrated that TM-EVLPs exerted sustained antiproliferative effects within the defined working concentration range.

### TM-EVLPs inhibit migration and invasion and modulate EMT-related markers

Following the antiproliferative effects observed *in vitro*, the impact of TM-EVLPs on motility and invasiveness was further examined. In wound healing assays, TM-EVLPs treatment noticeably delayed scratch closure in both A549 and H1975 cells compared with the control group, with wider residual gaps remaining at 24 h and 48 h. Quantification showed an overall dose-associated reduction in migration rate, with the most consistent inhibition at the high concentration, whereas the medium concentration showed a decreasing trend at some time points (Figures 2D,F). At the high-dose condition, the extent of wound-closure inhibition approached that observed in the cisplatin group, serving as an effect-size reference under the same *in vitro* conditions.

Consistent results were obtained in Transwell invasion assays. Relative to the control group, TM-EVLPs reduced the number of cells traversing the Matrigel-coated membrane in both cell lines, and the reduction was most consistent at the high concentration, whereas the lower concentrations showed a decreasing trend. High-dose TM-EVLPs produced an invasion-suppressive trend similar to cisplatin. This comparison served to contextualize the magnitude of TM-EVLP activity against a standard cytotoxic agent under the same *in vitro* conditions (Figures 2G,I). Together, these findings indicated that, beyond restraining proliferation, TM-EVLPs reduced the migratory and invasive potential of NSCLC cells.

**FIGURE 3:**
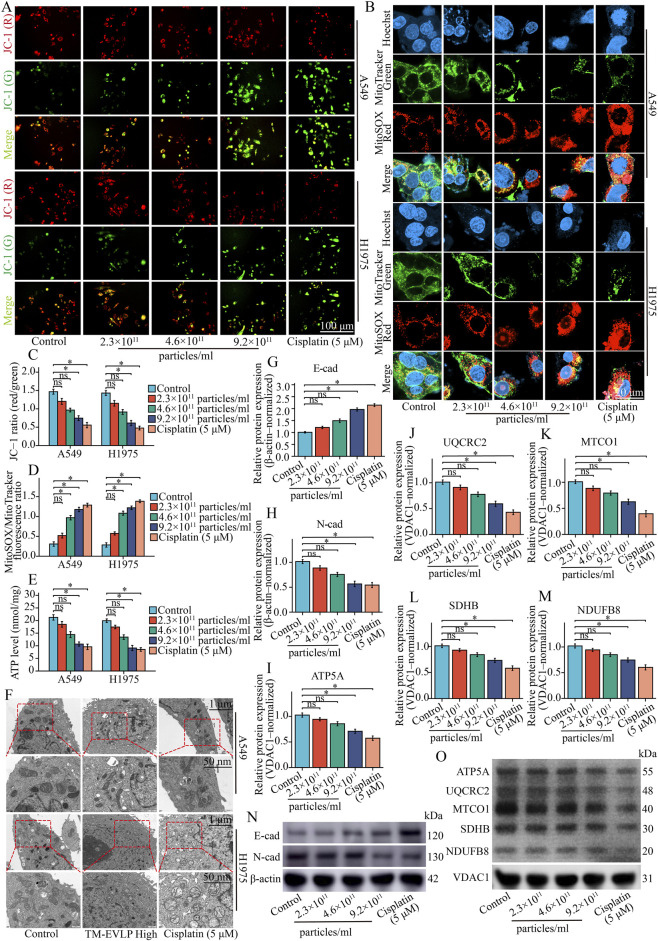
TM-EVLPs are associated with mitochondrial dysfunction and modulate EMT-related markers in NSCLC cells. **(A)** JC-1 staining in A549 and H1975 cells after TM-EVLPs treatment or cisplatin (5 μM). **(B)** Co-staining of MitoSOX and MitoTracker in A549 and H1975 cells following TM-EVLPs treatment or cisplatin (5 μM), with Hoechst nuclear counterstaining. **(C)** Quantification of the JC-1 red/green ratio. **(D)** Quantification of MitoSOX fluorescence intensity. **(E)** Intracellular ATP levels in A549 and H1975 cells after TM-EVLPs treatment or cisplatin (5 μM). **(F)** Transmission electron microscopy images showing mitochondrial ultrastructure in A549 and H1975 cells under control, TM-EVLPs, or cisplatin conditions. **(G–M)** Densitometric quantification of EMT-related proteins (E-cadherin and N-cadherin) and OXPHOS-related proteins (ATP5A, UQCRC2, MTCO1, SDHB, and NDUFB8). **(N)** Representative immunoblots of E-cadherin and N-cadherin with β-actin as the loading control. **(O)** Representative immunoblots of ATP5A, UQCRC2, MTCO1, SDHB, and NDUFB8 with VDAC1 as the mitochondrial loading control. TM-EVLPs, *Taraxacum mongolicum*-derived extracellular vesicle-like particles; NSCLC, non-small cell lung cancer; JC-1, mitochondrial membrane potential probe; OXPHOS, oxidative phosphorylation; EMT, epithelial–mesenchymal transition. *P < 0.05, ns, not significant. Data are presented as mean ± SEM (n = 3 independent experiments).

To provide molecular support for the observed phenotypic changes, EMT-related markers were evaluated. Western blotting and densitometric analysis showed increased E-cadherin and decreased N-cadherin after TM-EVLPs treatment, showing an overall dose-associated pattern, with more consistent changes observed at the high concentration (Figures 3G,H,N). Overall, the inhibition of migration and invasion by TM-EVLPs was accompanied by coordinated modulation of EMT-related markers.

### TM-EVLP treatment is accompanied by mitochondrial dysfunction and ultrastructural abnormalities

To explore a potential bioenergetic basis for the altered biological behavior of NSCLC cells, mitochondrial membrane potential was examined using JC-1 staining. Compared with the control group, TM-EVLPs treatment reduced the JC-1 aggregate associated red fluorescence and increased monomer associated green fluorescence in both A549 and H1975 cells, indicating depolarization of ΔΨm. Quantitative analysis showed an overall decline in the JC-1 red to green fluorescence ratio with increasing TM-EVLP concentrations (Figures 3A,C). At the highest dose, the reduction in ΔΨm approached the magnitude observed in the cisplatin-treated cells, serving as an effect-size reference rather than indicating mechanistic overlap.

Assessment of mitochondrial oxidative stress further supported these findings. MitoTracker Green enabled clear visualization of the mitochondrial network, whereas MitoSOX Red signals increased after TM-EVLP treatment, with stronger mitochondrial red fluorescence at the higher concentrations. Correspondingly, quantitative analysis showed an overall increasing trend in mitochondrial ROS-related signals across the TM-EVLP concentration range (Figures 3B,D).

Consistent with impaired mitochondrial bioenergetics, ATP quantification showed a dose-associated reduction in ATP levels in A549 and H1975 cells after TM-EVLPs treatment (Figure 3E), with more consistent decreases at the high dose and an overall decreasing trend at the medium dose; the lowest working dose showed a decreasing trend in A549 cells. Ultrastructural examination by TEM revealed that mitochondria in the control group largely retained intact morphology with relatively well-defined cristae. In contrast, high-dose TM-EVLPs induced pronounced morphological abnormalities characterized by compromised structural integrity and disorganized cristae. Similar ultrastructural damage was also observed in the cisplatin group (Figure 3F).

To further verify compromised mitochondrial respiration at the molecular level, mitochondrial respiratory chain related proteins were examined. Western blotting and densitometric analysis showed an overall downward trend in ATP5A, UQCRC2, MTCO1, SDHB, and NDUFB8 after TM-EVLP treatment, with more consistent reductions at the high concentration. Cisplatin also decreased these proteins, and the high-dose TM-EVLPs group exhibited a comparable trend (Figures 3I–M,O). Collectively, these data show that TM-EVLP treatment was accompanied by ΔΨm loss, increased mtROS, reduced ATP production, reduced OXPHOS protein abundance, and mitochondrial ultrastructural abnormalities *in vitro*, with the most consistent changes observed at the high concentration, supporting an association between anti-NSCLC phenotypes and mitochondrial structural/functional alterations.

### Oral TM-EVLPs suppress tumor growth *in vivo* with mitochondrial changes and acceptable histological safety

**FIGURE 4:**
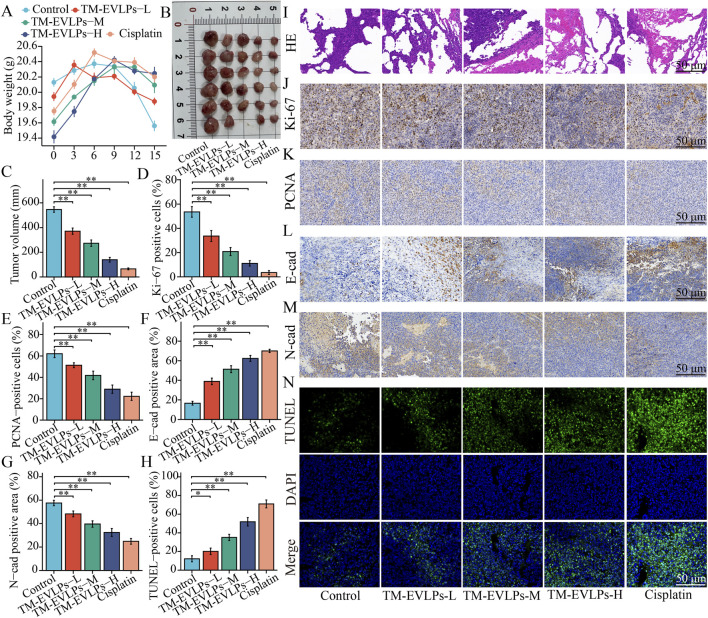
TM-EVLPs suppress NSCLC tumor growth *in vivo* and modulates proliferation- and EMT-related markers. **(A)** Body weight changes during treatment in tumor-bearing mice across the indicated groups. **(B)** Representative images of excised tumors. **(C)** Tumor volume at the study endpoint. **(D–H)** Quantification of Ki-67–positive cells **(D)**, PCNA-positive cells **(E)**, E-cadherin–positive area **(F)**, N-cadherin–positive area **(G)**, and TUNEL-positive cells **(H)** in tumor tissues. **(I)** Representative hematoxylin and eosin (H&E) staining of tumor sections. **(J–M)** Representative immunohistochemical staining of Ki-67 **(J)**, PCNA **(K)**, E-cadherin **(L)**, and N-cadherin **(M)** in tumors from each group. **(N)** Representative TUNEL staining (green) with DAPI nuclear counterstaining (blue) in tumor sections. TM-EVLPs, *Taraxacum mongolicum*-derived extracellular vesicle-like particles; NSCLC, non-small cell lung cancer; H&E, hematoxylin and eosin; PCNA, proliferating cell nuclear antigen; TUNEL, terminal deoxynucleotidyl transferase dUTP nick end labeling; DAPI, 4′,6-diamidino-2-phenylindole. *P < 0.05, **P < 0.01. Data are presented as mean ± SEM (n = 6).

In the xenograft model, TM-EVLPs were evaluated at predefined protein-normalized oral doses of 5, 10, and 20 mg/kg (low, medium, and high), and treatment reduced tumor burden in a dose-dependent manner. At the endpoint, tumors from the low, medium, and high TM-EVLPs groups were visibly smaller than those from the control group, and quantitative analysis of tumor volume confirmed an enhanced inhibitory effect with increasing doses. The antitumor trend in the high-dose TM-EVLPs group was comparable to that observed in the cisplatin group (Figures 4B,C). During the treatment period, body weight remained within a relatively stable range across groups, and no sustained body weight loss pattern was observed in the TM-EVLPs groups (Figure 4A).

Histological evaluation and proliferation-associated indices further supported the *in vivo* antitumor phenotype. H&E staining showed that, relative to the densely distributed tumor cells in the control group, TM-EVLPs treatment led to a looser tumor architecture with reduced cellular density. More frequent pale, acellular areas and cleft-like spaces were observed, suggesting expansion of necrosis-like regions. These changes were more prominent in the high-dose group and resembled those in the cisplatin group (Figure 4I). IHC analysis demonstrated reduced Ki-67 and PCNA positivity after TM-EVLPs treatment, with a progressive decline across the dose gradient (Figures 4D,E,J,K), indicating suppressed tumor cell proliferation. TUNEL staining revealed increased TUNEL positivity in TM-EVLPs-treated tumors, with an increasing trend as the dose rose (Figures 4H,N), consistent with elevated cell death; the magnitude of change in the high-dose group approached that in the cisplatin group. In addition, IHC for EMT-related markers showed increased E-cadherin positivity and decreased N-cadherin positivity following TM-EVLPs treatment, displaying an overall dose-associated pattern (Figures 4F,G,L,M). Together, these tissue-level findings support that the *in vivo* antitumor activity of TM-EVLPs aligns with reduced proliferation, enhanced cell death, and coordinated modulation of EMT-related markers.

**FIGURE 5:**
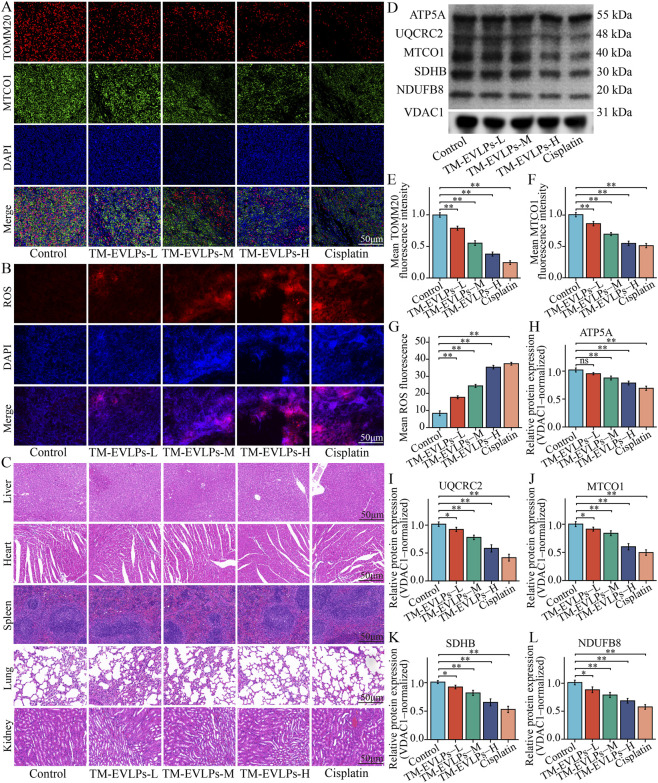
Tumor-tissue mitochondrial marker/ROS staining and organ histopathology following TM-EVLPs treatment. **(A)** Representative dual immunofluorescence images of TOMM20 (red) and MTCO1 (green) in tumor sections from each group, with DAPI nuclear counterstaining. **(B)** Representative fluorescence images of ROS staining in tumor sections, with DAPI counterstaining. **(C)** Representative H&E staining of liver, heart, spleen, lung, and kidney tissues from each group. **(D)** Representative immunoblots of OXPHOS-related proteins (ATP5A, UQCRC2, MTCO1, SDHB, and NDUFB8) in tumor tissues, with VDAC1 as the loading control. **(E–G)** Quantification of mean fluorescence intensity for TOMM20 **(E)**, MTCO1 **(F)**, and ROS **(G)** in tumor sections. **(H–L)** Densitometric quantification of ATP5A **(H)**, UQCRC2 **(I)**, MTCO1 **(J)**, SDHB **(K)**, and NDUFB8 **(L)** normalized to VDAC1. TM-EVLPs, *Taraxacum mongolicum*-derived extracellular vesicle-like particles; OXPHOS, oxidative phosphorylation; H&E, hematoxylin and eosin; ROS, reactive oxygen species; DAPI, 4′,6-diamidino-2-phenylindole. *P < 0.05, **P < 0.01, ns, not significant. Data are presented as mean ± SEM (n = 6).

Given that *in vitro* experiments indicated TM-EVLPs-associated mitochondrial injury, mitochondrial status and oxidative stress were further examined in tumor tissues. Dual immunofluorescence staining of MTCO1 and TOMM20 showed reduced signal intensities after TM-EVLPs treatment, with a progressive weakening across doses, consistent with fluorescence quantification (Figures 5A,E,F). ROS staining demonstrated increased ROS-related fluorescence signals in TM-EVLPs-treated tumors with an overall dose-dependent trend (Figures 5B,G). At the molecular level, Western blotting of tumor tissues showed that ATP5A, UQCRC2, MTCO1, SDHB, and NDUFB8 were downregulated after TM-EVLPs treatment, and the decrease became more pronounced with higher doses. The high-dose group exhibited a trend similar to the cisplatin group (Figures 5D,H–L).

For histological safety assessment, H&E staining of major organs, including liver, heart, spleen, lung, and kidney, did not reveal apparent structural disruption or marked inflammatory injury (Figure 5C). Together with the stable body weight and unremarkable general condition, these findings suggest no overt toxicity based on gross observation and organ histology under the current dosing and observation window.

## Discussion

Plant-derived EVLPs have emerged as a natural nanoscale platform capable of carrying diverse endogenous bioactive cargo. Their reported gastrointestinal stability and growing evidence base for oral delivery have sustained interest in exploring their utility for cancer intervention (Fang and Liu, 2022; Hao et al., 2024). However, oral administration does not necessarily translate into systemic bioavailability or tumor accumulation. Biodistribution studies of edible plant-derived nanovesicles frequently report predominant localization in the gastrointestinal tract and associated lymphoid tissues after gavage, whereas extra-intestinal exposure is variable and preparation-dependent (Kim and Kim, 2022). Despite this progress, the field continues to face common obstacles, including the absence of harmonized quality control criteria and an insufficient causal evidence chain linking specific cargo to mechanism of action in tumor-relevant settings (Zhao Q. et al., 2024). In this study, TM-EVLPs showed a consistent inhibitory trend on malignant phenotypes in NSCLC models, and the observed cellular changes aligned with mitochondrial functional readouts and ultrastructural alterations. This convergence suggests that TM-EVLPs engage a mitochondria-centered bioenergetic stress response characterized by disrupted mitochondrial homeostasis and an increased ROS burden, which accompanies the suppression of malignant phenotypes (Sessions and Kashatus, 2021; Du et al., 2025). Because cargo-level profiling and fractionation (e.g., RNA sequencing, proteomics, metabolomics, or depletion-based attribution tests) were not performed, this inference is restricted to an organelle-response signature and does not identify a single causal cargo entity.

From an ethnopharmacological reporting perspective, the present work distinguishes between current-batch physicochemical confirmation and previously established phytochemical characterization. In the current study, TM-EVLP preparations were directly confirmed by NTA, TEM, protein profiling, and protein quantification, whereas previously established LC–MS/MS fingerprinting data were cited to provide source-relevant compositional support for the Taraxacum mongolicum-derived vesicle preparation. Because TM-EVLPs are a vesicle-enriched particulate isolate rather than a conventional solvent extract, process-transparency parameters were reported to improve reproducibility without overstating newly generated phytochemical data in the present study.

From a novelty perspective, TM-EVLPs differ from many previously reported plant-derived EVLP systems in both source attributes and mechanistic focus. Many widely studied orally administered plant nanovesicles have been isolated from edible fruits/vegetables (e.g., grapefruit-derived nanovesicles) and were initially developed for gastrointestinal immune targeting or as delivery vehicles, where antitumor benefits are often discussed in the context of immune/microbiota modulation and/or exogenous drug loading rather than a defined organelle-centric vulnerability (Wang et al., 2014). In contrast, *Taraxacum mongolicum* is a pharmacopeial botanical drug, and emerging evidence for Taraxacum/dandelion-derived vesicles has largely centered on anti-infective/anti-inflammatory indications (Tan et al., 2024; Sun et al., 2025). Here, we extend botanical drug–derived EVLP research to an NSCLC setting and provide a mitochondria-centered organelle-response signature—OXPHOS suppression, ΔΨm collapse, mtROS elevation, and cristae disruption—linked to coordinated inhibition of proliferation and motility phenotypes. Importantly, the oral-gavage xenograft study recapitulated mitochondrial marker and OXPHOS protein changes in tumors, supporting a tumor-relevant mechanistic phenotype, although direct biodistribution/PK tracking remains to be established.

A prerequisite for TM-EVLPs to elicit downstream effects is the establishment of productive contact with NSCLC cells and successful intracellular delivery. Previous studies have shown that, after binding to the recipient cell surface, extracellular vesicles can enter the endosomal system through multiple uptake/entry routes, including clathrin-mediated endocytosis, caveolin-mediated endocytosis, macropinocytosis, and phagocytosis, and subsequently release protein, lipid, and nucleic acid cargo during membrane fusion events or through endosomal escape, thereby initiating intracellular signaling cascades (Liu and Wang, 2023; Kim et al., 2024). Plant-derived EVLPs often retain surface features such as plant membrane lipids and glycosylation patterns. These physicochemical characteristics can influence membrane affinity and may also shape the preferred endocytic route and intracellular trafficking efficiency in tumor cells (Yi et al., 2023; Zhang et al., 2024). In the fluorescence-labeling assay of this study, discrete cell-associated fluorescent signals were observed after incubation and washing, supporting productive contact between TM-EVLPs and NSCLC cells as a prerequisite for downstream effects.

Following intracellular delivery, the suppression of malignant phenotypes by TM-EVLPs aligns with a mitochondria-centered bioenergetic stress response together with an increased ROS burden. Beyond serving as the primary source of ATP and essential metabolic intermediates, mitochondria function as a central hub that integrates cell fate signaling under therapeutic stress (Shen et al., 2022). In this study, the loss of ΔΨm accompanied by elevated mitochondrial reactive oxygen species (mtROS), reduced ATP levels, and downregulation of key OXPHOS complex subunits, together with cristae disorganization at the ultrastructural level, forms a coherent structure-to-function evidence chain. This pattern is consistent with an energy crisis state induced by reduced respiratory chain efficiency and increased electron leakage (Molina et al., 2018). Importantly, NSCLC is characterized by pronounced metabolic plasticity and does not rely exclusively on glycolysis. OXPHOS activity can make a substantial contribution to sustaining proliferation and adapting to treatment pressure. Accordingly, impaired OXPHOS may restrict cell growth by limiting energy production and biosynthetic substrate supply while amplifying oxidative stress, thereby eroding the survival advantages of tumor cells (Sun et al., 2023). In parallel, antitumor strategies targeting OXPHOS are being actively developed and evaluated in clinical settings, yet the therapeutic window and toxicity boundaries of respiratory chain inhibition have been repeatedly emphasized (Cadassou and Jordheim, 2023; Machado et al., 2023). These considerations indicate that a bioenergetic constraint framework not only rationalizes the antiproliferative effects observed here, but also provides a foundation for discussing how mitochondrial stress may couple to reduced migration and invasion and to diminished EMT-related plasticity. Mechanistic causality can be further strengthened by interventional epistasis experiments, including testing whether ROS scavengers attenuate TM-EVLP–induced mtROS accumulation and restore ΔΨm/ATP, whether OXPHOS inhibitors/uncouplers phenocopy the mitochondrial stress signature, and whether mitochondrial rescue agents reverse key phenotypes. Because cargo attribution and non-malignant bronchial epithelial comparisons were not performed, we interpret these findings as an organelle-response signature and working mechanistic model, rather than definitive proof of a single causal cargo or tumor selectivity.

The maintenance of migratory and invasive behavior and EMT-associated phenotypes depends not only on transcriptional program switching but also on sustained mitochondrial energy supply. Previous studies have shown that, during tumor cell migration, mitochondria can undergo spatial redistribution and accumulate at the leading edge to meet the local demand for ATP and metabolic substrates required for cytoskeletal remodeling and adhesion turnover. In parallel, ROS-linked signaling contributes to the regulation of cell motility and phenotypic plasticity (Crosas-Molist et al., 2023; Mosier et al., 2024). The hub role of mitochondria in metastasis has also been systematically summarized, encompassing respiratory chain–supported bioenergetics, mitochondrial dynamics, and mitochondrial stress signaling in shaping invasive cascades (Chen et al., 2024; Zhou et al., 2024). In the present study, reduced migration and invasion accompanied by increased E-cadherin and decreased N-cadherin is consistent with a framework in which constrained mitochondrial bioenergetics weakens the energetic and anabolic support needed for motile phenotypes, including processes such as membrane lipid production. At the same time, elevated mtROS may destabilize mesenchymal-like states by perturbing EMT-associated regulatory networks (Liaghat et al., 2024). Notably, ROS exerts context- and dose-dependent biphasic effects on EMT and metastasis. Moderate ROS levels can promote migration, whereas a sustained high ROS burden is more likely to restrict invasion and favor oxidative stress–associated cell death (An et al., 2024), which aligns with the overall suppression of malignant phenotypes observed in this study.

*Taraxacum mongolicum* has a chemically complex profile, and multiple metabolite classes, including triterpenoids, phenolic acids, and flavonoids, have shown convergent inhibitory signaling across diverse tumor models. Within these mechanisms, mitochondrial bioenergetics and ROS signaling have frequently emerged as important functional nodes (Yan et al., 2024; Wang A. et al., 2025). Against this background, TM-EVLPs may represent a functional fraction that delivers endogenous, multidimensional bioactivity from *Taraxacum mongolicum* in a membranous particulate form, such that intracellular effects reflect the integration of composite cargo rather than dominance by a single molecule. Studies on plant-derived EVLPs indicate that these particles can concurrently carry lipids, proteins, nucleic acids, and secondary metabolites and access the recipient endosomal system, thereby enabling coordinated, multi-pathway modulation at the organelle level (Zhang et al., 2024; 2025). Evidence from vesicle-like particles of other plant origins in tumor models is also consistent with this view, showing that increased ROS burden coupled with mitochondrial perturbation can simultaneously restrain proliferation, migration, and invasion (Chen et al., 2022). Accordingly, interpreting the anti-NSCLC activity of TM-EVLPs as a composite cargo–driven mitochondrial stress program that converges malignant phenotypes onto a shared mechanistic axis is consistent with the explanatory scope supported by the present results and with biological coherence.

Although the specific active cargo of TM-EVLPs was not resolved here, plant-derived EVLPs are commonly enriched in membrane phospholipids (e.g., phosphatidic acid, phosphatidylethanolamine, and phosphatidylcholine) that may affect vesicle uptake and stress signaling in recipient cells (Huang et al., 2025). In addition, small RNAs including miRNA-like species have been reported in vesicles from several edible plants (Urbanelli et al., 2025), suggesting a potential nucleic-acid cargo layer that warrants profiling in TM-EVLPs. From the source botanical drug, Taraxacum species contain abundant phenolic-acid, flavonoid, and triterpenoid metabolites with reported redox- and mitochondria-related activities (Yan et al., 2024); determining whether such metabolites are vesicle-associated or co-isolated fractions will be important for establishing causality.

In NSCLC, mitochondria not only supply bioenergetic output and metabolic substrates but also help define the cellular tolerance threshold to therapeutic pressure through membrane potential maintenance, respiratory chain electron transfer efficiency, and ROS signaling (Li et al., 2023; Wang et al., 2024). A decline in mitochondrial function can constrain cell cycle progression and weaken anabolic support, whereas an increased mtROS burden can reshape stress-associated transcriptional programs and proteostasis pathways. Together, these changes reduce the capacity of tumor cells to sustain the highly plastic state required for migration and invasion. As a result, a single upstream stressor may concurrently manifest as both proliferative restriction and attenuation of invasive phenotypes (Guo et al., 2020; Parlani et al., 2023). Within this biological framework, TM-EVLPs can be viewed as a natural nanoscale unit in which composite cargo acts in concert, producing an additive pressure pattern at the organelle level and rendering mitochondria a preferentially vulnerable node (Krieg et al., 2024). Once mitochondrial compensatory capacity is exceeded, constrained energy supply and elevated ROS typically co-emerge and amplify shifts in cell fate signaling, thereby diminishing tumor cell survival and expansion advantages and driving an overall suppression of malignant behavior (Nakasuka et al., 2021). Given the concordant changes observed in this study across phenotypic outcomes, mitochondrial functional readouts, and ultrastructural features, the anti-NSCLC activity of TM-EVLPs can be summarized as a mitochondrial stress-linked process that converges on multiple phenotypic endpoints. This interpretation also provides a testable mechanistic hypothesis and histology-supported rationale for subsequent, more refined validation of key cargo and causal mechanism chains.

Although this study systematically delineated the anti-NSCLC effects of TM-EVLPs using phenotypic assays together with mitochondrial functional and ultrastructural readouts, several issues warrant further clarification. First, cargo-level profiling and fractionation were not performed; therefore, the causal vesicular entity (membrane lipids, intraluminal proteins/RNAs, vesicle-associated metabolites, and/or residual non-vesicular co-isolates) and direct molecular targets cannot yet be specified. Our data currently support an integrated, composite-cargo mode of action that manifests as an organelle-level mitochondrial stress phenotype rather than a single-metabolite causal chain. Second, the work relied mainly on A549/H1975 cells and a nude-mouse xenograft model, and thus may not fully capture mitochondrial stress heterogeneity across NSCLC subtypes or under immunocompetent microenvironmental contexts. Third, the temporal ordering among mitochondrial impairment, oxidative burden, and EMT-associated changes requires refinement through dynamic tracking and interventional experiments (e.g., ROS scavenging, OXPHOS perturbation, and mitochondrial rescue approaches). Fourth, normal bronchial epithelial cell controls were not evaluated; thus, cancer selectivity and potential mitochondrial toxicity in non-malignant airway epithelium remain to be determined. Together with the stable body weight and unremarkable general condition, these findings suggest no overt toxicity based on gross observation and organ histology under the current dosing and observation window. Future *in vivo* studies will incorporate serum biochemical indices, including alanine aminotransferase (ALT), aspartate aminotransferase (AST), and creatinine, to quantitatively evaluate hepatic and renal safety and further strengthen the oral safety profile. In addition, the oral stability, systemic absorption, and tumor biodistribution of intact TM-EVLPs were not directly tracked; therefore, the *in vivo* data support a tumor-relevant phenotype but do not establish vesicle-level pharmacokinetics. Fifth, because TM-EVLPs are heterologous (cross-kingdom) vesicles administered to mammals, potential risks must be considered, including context-dependent immunogenicity, altered biodistribution/clearance, and quality-related concerns such as residual co-isolates or inadvertent microbial contaminants; these issues highlight the need for standardized purity criteria and systematic safety evaluation to support translation (Bai et al., 2025; Li et al., 2026). Despite these limitations, by converging TM-EVLP activity onto a testable organelle-level mechanism centered on constrained mitochondrial bioenergetics, this study provides a clear framework for subsequent mechanistic dissection and translational de-risking.

## Conclusion

This study demonstrates that TM-EVLPs suppress both proliferation and migration and invasion in NSCLC models and are accompanied by a shift of EMT-associated markers toward an epithelial phenotype. Multi-level evidence indicates that the antitumor activity is closely associated with mitochondrial structural alterations and constrained bioenergetic capacity, characterized by loss of ΔΨm, increased mtROS burden, and downregulation of OXPHOS-related proteins, with functional readouts aligning with ultrastructural observations. The *in vivo* xenograft experiments further support the tumor-suppressive potential of TM-EVLPs and showed no apparent histological abnormalities in major organs under the conditions tested. Taken together, TM-EVLPs restrain NSCLC malignant phenotypes in association with a mitochondria-centered stress signature. Comparative evaluation in non-malignant bronchial epithelial models will be required to define the cellular selectivity and therapeutic window of TM-EVLPs. Definitive identification of the responsible cargo layer and causal validation will require dedicated cargo profiling and fractionation in future work.

## Data Availability

The original contributions presented in the study are included in the article/Supplementary Material, further inquiries can be directed to the corresponding author.

## Supplementary material

SUPPLEMENTARY TABLE S1 Key reagents, kits, consumables, and biological resources used in this study.

SUPPLEMENTARY TABLE S2 Primary and secondary antibodies used for Western blotting, immunohistochemistry, and immunofluorescence in this study.
